# Recent Advances in the Pathogenesis of Autoimmune Hair Loss Disease Alopecia Areata

**DOI:** 10.1155/2013/348546

**Published:** 2013-09-18

**Authors:** Taisuke Ito

**Affiliations:** Department of Dermatology, Hamamatsu University School of Medicine, 1-20-1 Handayama, Higashi-ku, Hamamatsu 431-1192, Japan

## Abstract

Alopecia areata is considered to be a cell-mediated autoimmune disease, in which autoreactive cytotoxic T cells recognize melanocyte-associated proteins such as tyrosinase. This review discusses recent advances in the understanding of the pathogenesis of alopecia areata, focusing on immunobiology and hormonal aspects of hair follicles (HFs). The HF is a unique “miniorgan” with its own immune and hormonal microenvironment. The immunosuppressive milieu of the anagen hair bulb modulated by immunosuppressive factors is known as “hair follicle immune privilege.” The collapse of the hair follicle immune privilege leads to autoimmune reactions against hair follicle autoantigens. Alopecia areata is sometimes triggered by viral infections such as influenza that causes excess production of interferons (IFN). IFN-**γ** is one of the key factors that lead to the collapse of immune privilege. This paper reviews the interactions between the endocrine and immune systems and hair follicles in the pathogenesis of alopecia areata.

## 1. Introduction 

### 1.1. Clinical Features

Alopecia areata (AA) is a relatively common disease encountered by dermatologists. Patients with AA represent approximately 0.7% to 3.8% of all the patients attending dermatology clinics [1, 2]. Males and females are affected equally [3]. Disease onset before the 4th decade was reported to be 85.5% in the Asian population [2]. 

Based on the extent of hair loss, the hair loss pattern of AA can be described as single delimited patches; patchy AA, in which there is a partial loss of scalp hair; alopecia totalis (AT), in which 100% of scalp hair is lost; or alopecia universalis (AU), in which there is a 100% loss of all scalp and body hair [4]. Less common types of AA include reticular patches of hair loss; ophiasis type, a band-like hair loss in the parieto-temporo-occipital area; ophiasis inversus (sisaipho), a rare band-like hair loss in the frontal parietotemporal scalp; and a diffuse thinning of a part or all of the scalp [4]. Another variant named “acute diffuse and total alopecia” is characterized by acute scalp hair loss, extensive involvement with infiltration of eosinophils around hair follicles (HFs), and a favorable prognosis. It was first described by Sato-Kawamura et al. and was thought to be limited to females [5], but Lew et al. described that is affected male patients in their case series [6].

### 1.2. Association with Other Autoimmune Diseases

AA may be associated with other autoimmune diseases, particularly thyroid autoimmune disease such as Hashimoto's thyroiditis and Basedow's disease. The prevalence of thyroid disease in patients with AA ranges from 8% to 28% [7]. The presence of thyroid autoantibodies does not correlate with AA severity [8], and treatment is not warranted. Vitiligo, an autoimmune skin disease affecting melanocytes, is also associated with AA. The prevalence of vitiligo in AA patients is 3% to 8% compared with 1% in the United States population [9]. These disease associations suggest a relationship between autoimmunity and AA.

### 1.3. Psychiatric Morbidity

AA is associated with psychiatric morbidity, especially anxiety and depression [10]. In a study of 31 AA patients, Colón et al. reported that 74% were given one or more lifetime psychiatric diagnoses based on structured psychiatric interviews [11]. The lifetime prevalence rates of major depression (39%) and generalized anxiety disorder (39%) were particularly high [11]. These studies indicate that patients with AA are at an increased risk for developing anxiety and depression, and psychiatric referral may be warranted in AA patients.

### 1.4. Histopathology

The accumulation of mononuclear cells in and around hair bulbs—so-called “swarm of bees”—is the most characteristic histopathological change in AA [12]. This is observable particularly in the acute stage of the disease, and it is composed of both CD4+ and CD8+ cells with a high CD4+/CD8+ ratio in clinically active disease [13]. In the chronic stage, there is a marked HF miniaturization and cell accumulation decreases, but accumulation of CD8+ T cells is still observable [14].

## 2. Pathogenesis

### 2.1. Genetics

Genetics play an important role in the pathogenesis of AA. For example, monozygotic twins who suffered from AA after mumps infection had a similar disease onset and hair loss patterns [15]. Specific alleles such as DQB1*03 and DRB1*1104 have been reported as markers of susceptibility to AA [16–20]. The HLA alleles DRB1*1104 (HLA-DR11) and DQB1*0301 (HLA-DQ7) may be associated with AT/AU [17]. These findings suggest that the onset and progression of AA is associated with specific HLA class II alleles [18–21]. Recently, Petukhova et al. [22] performed a genome-wide association study (GWAS) to determine the genetic architecture of AA in a sample of 1,054 AA cases and 3,278 controls using a combination of Illumina 610 K and 550 K arrays. The GWAS revealed 139 single nucleotide polymorphisms (SNPs) that are significantly associated with AA (*P* ≤ 5 × 10^−7^). Several susceptibility loci for AA were identified, most of which were clustered in eight genomic regions and fell within discrete linkage disequilibrium blocks. These include loci on chromosome 2q33.2 containing *CTLA4*, chromosome 4q27 containing *IL-2/IL-21*, chromosome 6p21.32 containing the *HLA*, chromosome 6q25.1 harboring the *ULBP* genes, chromosome 10p15.1 containing *IL-2RA (CD25)*, and chromosome 12q13 containing *Eos (IKZF4)* and *ERBB3*. One SNP resides on chromosome 9q31.1 within syntaxin 17 (*STX17*), and one resides on chromosome 11q13, upstream from peroxiredoxin 5 (*PRDX5*). The *ULBP* genes encoding the ligands of the natural killer cell receptor NKG2D reside in a 180-kilobase MHC class I-related cluster on human chromosome 6q25.1. NKG2D is expressed on natural killer (NK) and CD8+ T cells, and is activated by ULBP. The role of the NK cell activating receptor, NKG2D, in the pathogenesis AA will be described later in this review.

### 2.2. Collapse of Hair Follicle Immune Privilege

One of the most intriguing features of hair biology is the immune privilege of the anagen HF [23, 24] that is characterized by an immunosuppressive milieu around the hair bulb. Apart from the anagen HF, other sites of immune privilege include the anterior eye chamber, parts of testis and ovaries, adrenal cortex, parts of the central nervous system enclosed by the blood-brain barrier, the placenta, and the hamster cheek pouch [23, 24]. The unique microenvironment of these immune-privileged sites protects the organs from deleterious immune reactions and loss of function. For example, severe inflammation of the anterior chamber of the eye could lead to blindness, and an immune reaction in the central nervous system could cause serious brain damage. Although scalp and trunk hair are not necessary for human survival, significant hair loss could be deadly for mammals such as polar bears, seals, and reindeer [25].

**Figure 1:**
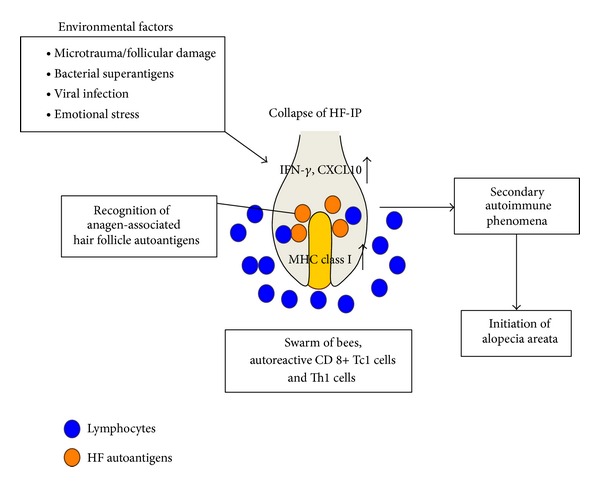
The pathogenesis of alopecia areata and treatment strategies. Environmental factors such as viral infections and bacterial superantigens may induce IFN-*γ* and CXCL10 expressions in the hair bulbs. Subsequently, autoreactive Th1 and Tc1 cells (blue circles) accumulate in and around hair bulbs—the so-called “swarm of bees.” Anagen-associated hair follicle (HF) autoantigens (orange circles) are recognized by Th1 and Tc1 cells, which lead to a secondary autoimmune phenomenon and resultant hair loss.

The HF immune privilege (IP) is maintained by several factors, including the lack of major histocompatibility complex (MHC) class I in the proximal outer root sheath (ORS) and matrix cells (Table 1). HFIP is present during anagen but is lost during the resting (telogen) and regression (catagen) phases of the hair cycle. Collapse of HFIP is thought to contribute to the development of AA (Figure 1), in which pigment-producing anagen hair bulbs are attacked by inflammatory cells [23]. MHC class I is strongly expressed in AA lesions, raising the possibility that CD8+ T cells react to autoantigens by binding to MHC class I molecules. Interferon (IFN)-*γ*, a key cytokine implicated in the pathogenesis of AA, upregulates MHC class I expression in cultured HFs in vitro [26]. Virus infections can increase the production of IFN-*γ* in vivo, and it has been reported that the swine flu virus can trigger or exacerbate AA [27]. 

### 2.3. Autoantigens

Several studies of AA suggest that melanogenesis-associated peptides expressed by melanin-producing anagen HFs are the key autoantigens targeted by autoreactive cytotoxic T cells [28, 29]. Possible involvement of melanogenesis-associated autoantigens in AA was suggested by the following observations: sparing of white/greying HFs in AA; regrowing hair shafts are usually white followed by repigmentation, association with vitiligo, and the sudden onset of fulminant AA affecting mostly pigmented HFs (overnight greying). Follicular melanocytes are possible targets in AA. Indeed, follicular melanocytes in AA show both histological and ultrastructural abnormalities [30]. Using the human scalp explant/SCID mouse transfer model, Gilhar et al. demonstrated that melanocyte-associated T cell epitopes are capable of functioning as autoantigens and result in AA in human scalp grafts [29]. Melanocyte HLA-A2-restricted peptides can activate T cells for the transfer of AA to autologous scalp skin grafts on SCID mice, indicating that melanocyte-associated autoantigens can be pathogenic.

### 2.4. “Swarm of Bees” Linked to Th1 Cytokines, Chemokines, and Chemotaxis

A unique histopathological feature in the acute stage of AA is the dense accumulation of lymphocytes around the hair bulbs—so-called “swarm of bees”—that is a result of the collapse of HFIP with exposure of autoantigens, leading to an accumulation of autoreactive T cells [12]. The mononuclear cells that accumulate in and around the lesional hair bulb consist of 60–80% CD4+ T cells, 20–40% CD8+ T cells, and NK cells [13, 31]. IFN-*γ*, a representative Th1 cytokines, is prominently expressed in AA lesions and may induce the collapse of HFIP by upregulating MHC class I expression [32]. Autoimmune hair loss similar to AA can be reproduced in C3H/HeJ mice by injecting IFN-*γ*, which induces the follicular expression of MHC classes I and II [33].

Several studies have examined the expression of chemokines and their receptors in AA. For example, the expression of Th1 chemokines CXCL9/MIG and CXCL10/IP-10 is increased in AA lesions [34, 35]. Serum CXCL9 is elevated in AA patients, and its level is correlated with disease activity [36]. Transcriptional profiling revealed that CXCL10 is highly upregulated in AA lesions compared with nonlesional skin [37]. Our study showed that the proportions of CXCR3+ CD4+ Th1 cells and CXCR3+ CD8+ Tc1 cells are significantly increased in the PBMCs of AA patients [11]. CD8+ T cells can be differentiated into two effector phenotypes, Tc1 and Tc2, which secrete different cytokines [38–40]. CD8+ T cells that secrete IFN-*γ* but not IL-4 and IL-5 are known as type I CD8+ cytotoxic T (Tc1) cells, while those that secrete IL-4 and IL-5 but not IFN-*γ* are type II CD8+ cytotoxic T (Tc2) cells. Tc1 cells kill tumor targets by either perforin or Fas-mediated mechanisms, whereas Tc2 cells mainly use the perforin pathway [41]. Tc1 cells are implicated in the development of autoimmune diseases such as experimental autoimmune thyroiditis [42]. In the acute phase AA, the proportion of MAGE-A3 is specific, IFN-*γ*-producing T cells in PBMCs is increased [11]. Therefore, the increased numbers of CXCR3+ CD8+ Tc1 cells may contribute to the cell-mediated autoimmune reactions in AA.

In addition, we demonstrated that freshly isolated CD4+ and CD8+ T cells from AA patients displayed strong chemotactic activity towards CXCL10 using a real-time chemotaxis assay [14]. The T cell chemotactic activity may be a result of activation. Considering the accumulation of lymphocytes in the acute phase of AA, our T cell chemotaxis results are consistent with the histopathological findings of AA in the acute phase [14].

### 2.5. NKG2D in Alopecia Areata

NKG2D is expressed not only in NK cells but also in CD8+ and gamma delta T cells [43, 44]. NKG2D recognizes MHC class I chain-related proteins MICA and MICB on target cells. NKG2D also recognizes surface glycoproteins that bind human cytomegalovirus UL16 proteins (ULBPs), from ULBP1 to ULBP6 (total of eight human ligands), which stimulates immune cells to attack target cells [45].

 NK cells have become a recent focus of AA research. The absent or low expression of MHC class I in HFs raises the question of how self/nonself-discrimination and self-tolerance are maintained [46]. As NK cells recognize and eliminate cells with an absent or low expression of MHC class I [27, 47–49], it is remarkable that very few NK cells gather around the MHC class I negative human anagen HFs [50]. Just like other healthy tissues, human anagen HFs in situ lack MICA expression [31, 51–53], which may explain why normal HFs are not subject to NK cell attack. However, in AA lesions, infiltrating CD56+ NK cells and CD8+ T cells prominently express NKG2D, and proximal ORS strongly expresses MICA, thus infiltrating NKG2D+ cells attack MICA-positive HFs [31]. Compared with normal controls and patients with other chronic inflammatory skin diseases such as atopic dermatitis, CD56+ NK cells and CD8+ T cells of AA patients have increased NKG2D expression. In addition, the percentage of NK cells that do not express NK cell-inhibitory KIR2DL2 and KIR2DL3 is significantly increased in AA patients compared with healthy controls [31]. A recent study of 20 families with AA from the United States and Israel has identified genes that may be associated with AA and other autoimmune diseases, such as *ULBP* genes that encode ligands for activating NKG2D [43].

In summary, immune-privileged, MHC class I-negative HFs are protected from NK cell attack by MICA-negative ORS, low expression of NKG2D on NK cells, and inhibitory KIRs. However, collapse of HFIP leads to NKG2D+ NK cell attack, and autoreactive NKG2D+ CD8+ T cells recognize autoantigens that result in apoptotic responses and hair loss in AA.

### 2.6. Stress Hormone and Alopecia Areata

To maintain homeostasis, the skin must respond to stressors such as ultraviolet light, mechanical injury, and chemical and biological insults. Emotional stress may also perturb skin homeostasis. Components of the hypothalamic-pituitary-adrenal (HPA) axis are present in the skin and are involved in the local response to stress [54]. As part of the HPA axis, corticotropin-releasing hormone (CRH) is a key stress-induced hormone that is present in the human HF. Specifically, CRH and CRH-1 receptors (CRH-R1) are expressed throughout the ORS [55], and CRH and CRH-R1 gene transcriptions occur in the human hair bulb. The human HF has a fully functional peripheral equivalent of the HPA axis, which may be involved in the stress response of the skin [56]. Organ culture human HFs secrete cortisol in response to CRH and possess feedback systems [56]. 

Using in situ hybridization, a clinical report examined CRH receptor expression in three AA patients who had experienced a significant emotional stress prior to hair loss. Skin from the affected scalp areas of all three patients showed an intense signal for CRH-2*β*. Samples from unaffected scalp areas of the same patients or from healthy controls showed only a weak background signal for the receptor [57]. The expression of CRH, ACTH, and *α*-MSH was significantly increased in the epidermis, HFs, and sebaceous glands of samples from AA patients compared with those from healthy controls. These results suggest the presence of an active neurogenic system and local HPA activity in AA lesions [57]. Immune activation not only affects CRH expression but also HPA activity [58–64].

Central and peripheral HPA activity under basal and stressful conditions were investigated in normal and AA-affected C3H/HeJ mice [65]. In response to psychological stress, normal mice showed marked plasma corticosterone elevation, whereas AA-affected mice showed no significant changes in corticosterone levels, suggesting that AA-affected mice have a blunted response to acute physiological stress. 

In conclusion, emotional stress may affect AA patients because of a blunted corticosterone response to hormones and immunological damage, and altered HPA activity may occur as a consequence of the immune response associated with AA.

## 3. Conclusion

The HF is a dynamic “miniorgan” with unique immune and hormone microenvironments. Immune privilege is the most intriguing feature of HF immunology that is characterized by the downregulation of MHC class I. The collapse of HFIP is induced by certain environmental factors such as virus infections, and it involves the production of the Th1/Tc1 chemokine, CXCL10, in HFs that attracts Th1 and Tc1 cells towards the hair bulbs. Consequently, HF autoantigens are recognized by autoreactive cytotoxic T cells. HPA activity in the HF links the immune and hormonal aspects of the HF. Environmental stress may influence both the immune and hormonal microenvironments of the HF and result in the development of AA. Patients with AA may have a blunted response to an acute physiological stressor, resulting in a reduced expression of glucocorticoids.

Recent advances in the understanding of the pathomechanism of AA may lead to the development of novel treatments for AA in the future.

## Figures and Tables

**Table 1:** The mechanism of HF immune privilege.

(i) Absent or barely detectable expression of MHC class I.	
(ii) Hair follicular melanocytes of the human anagen scalp are MHC class I-negative.	
(iii) Downregulation of the MHC class I pathway-related molecules *β*2-microglobulin and transportation of antigen processing-2 (TAP-2).	
(iv) Downregulation of interferon regulatory factor-1 expression.	
(v) Upregulation of immunosuppressive factors such as TGF-*β*1 and TGF-*β*2, ACTH, and *α*-MSH.	
(vi) Absence of MHC class II+ or NLDC-145+ Langerhans cells.	
(vii) Sparse distribution of NK cells and CD4+ and CD8+ T cells.	
(viii) Absence of lymphatics.	
